# Catalytically-active inclusion bodies for biotechnology—general concepts, optimization, and application

**DOI:** 10.1007/s00253-020-10760-3

**Published:** 2020-07-10

**Authors:** Vera D. Jäger, Robin Lamm, Kira Küsters, Gizem Ölçücü, Marco Oldiges, Karl-Erich Jaeger, Jochen Büchs, Ulrich Krauss

**Affiliations:** 1 Institut für Molekulare Enzymtechnologie, Heinrich-Heine-Universität Düsseldorf, Forschungszentrum Jülich GmbH, 52425 Jülich, Germany; 2 Bioeconomy Science Center (BioSC), c/o Forschungszentrum Jülich, Jülich, 52425 Germany; 3 Present Address: Department of Bioproducts and Biosystems, Aalto University, Kemistintie 1, Espoo, 02150 Finland; 4 AVT-Chair for Biochemical Engineering, RWTH Aachen University, Aachen, 52074 Germany; 5 Institute of Bio- and Geosciences IBG-1: Biotechnology, Forschungszentrum Jülich GmbH, Jülich, 52425 Germany; 6 Institute of Biotechnology, RWTH Aachen University, 52074 Aachen, Germany

**Keywords:** Catalytically active inclusion bodies, Enzyme immobilization, Protein engineering, Synthetic biology, Protein co-localization, Biocatalysis, Synthetic reaction cascades, Upstream and downstream processing

## Abstract

**Abstract:**

Bacterial inclusion bodies (IBs) have long been considered as inactive, unfolded waste material produced by heterologous overexpression of recombinant genes. In industrial applications, they are occasionally used as an alternative in cases where a protein cannot be expressed in soluble form and in high enough amounts. Then, however, refolding approaches are needed to transform inactive IBs into active soluble protein. While anecdotal reports about IBs themselves showing catalytic functionality/activity (CatIB) are found throughout literature, only recently, the use of protein engineering methods has facilitated the on-demand production of CatIBs. CatIB formation is induced usually by fusing short peptide tags or aggregation-inducing protein domains to a target protein. The resulting proteinaceous particles formed by heterologous expression of the respective genes can be regarded as a biologically produced bionanomaterial or, if enzymes are used as target protein, carrier-free enzyme immobilizates. In the present contribution, we review general concepts important for CatIB production, processing, and application.

**Key points:**

*• Catalytically active inclusion bodies (CatIBs) are promising bionanomaterials.*

*• Potential applications in biocatalysis, synthetic chemistry, and biotechnology.*

*• CatIB formation represents a generic approach for enzyme immobilization.*

*• CatIB formation efficiency depends on construct design and expression conditions.*

## Introduction

**Fig. 1:**
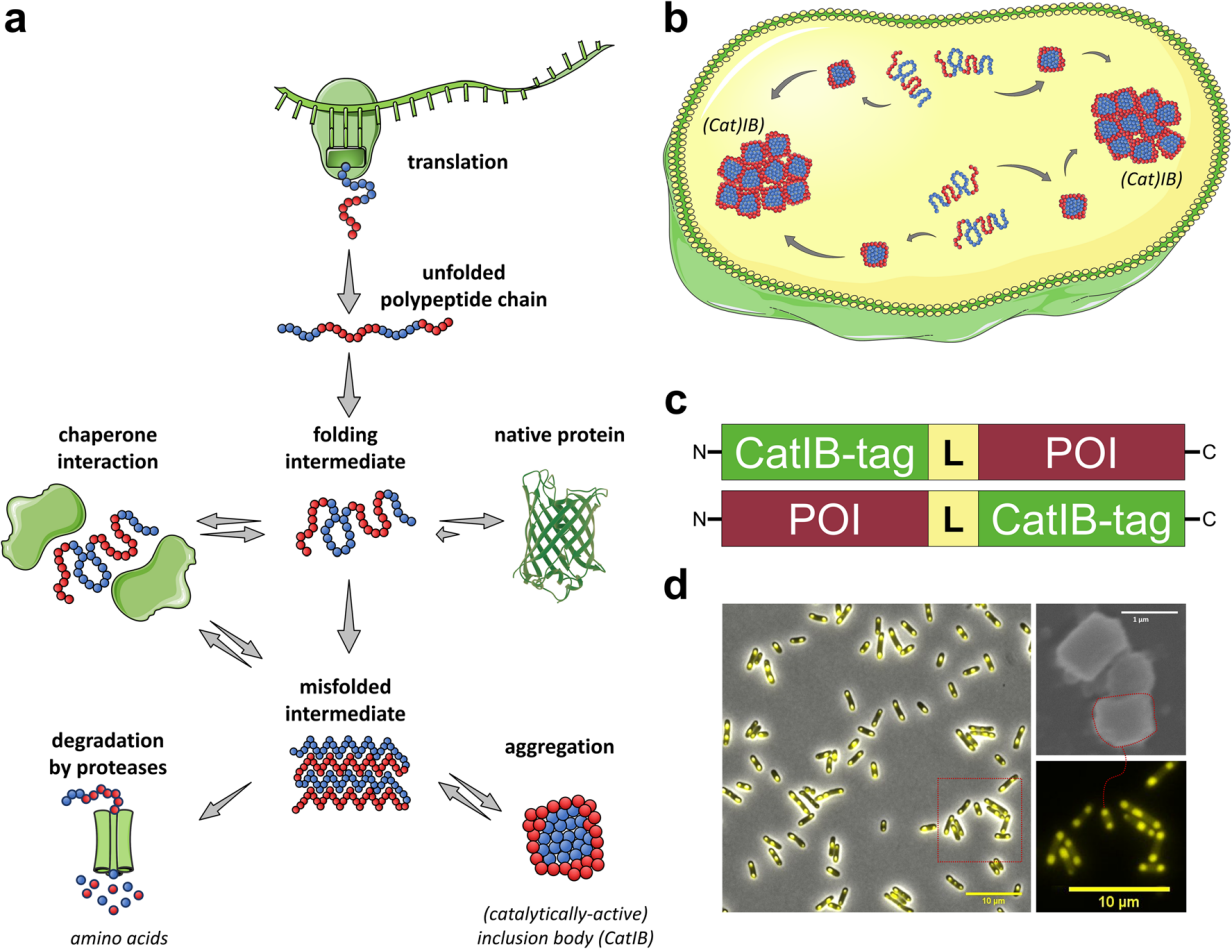
(Cat)IB formation in bacteria. **a** Cellular processes leading to the formation of inclusion bodies (IBs), which are subsequently **b** deposited at the cell poles likely driven by nucleoid exclusion (Rinas et al. 2017; Kopito 2000). Structural regions that adopt a native or native-like fold are shown as red-filled circles. Aggregation-prone sequence stretches are depicted as blue-filled circles. **c** Fusion protein architectures for the induction of CatIB formation. In all cases, an aggregation-inducing CatIB-tag is fused either N- or C-terminally to a protein of interest (POI). To link both protein modules, usually linker polypeptides (L) of variable length are used. **d** Overlay of phase-contrast and fluorescence microscopy image of TDoT-L-YFP producing *E. coli* BL21(DE3) cells (Jäger et al. 2019a). The lower right panel depicts a close-up view to better visualize polar localization of the produced CatIBs. The upper right panel depicts a scanning electron microscopy image of isolated CatIB particles

Bacteria such as *Escherichia coli* often produce inclusion bodies (IBs) as consequence of the accumulation of misfolded protein due to strong overexpression of heterologous genes (Baneyx and Mujacic 2004). For a long time, IBs have thus been regarded as inactive waste or, at best, as by-products consisting solely of misfolded and aggregated proteins. Due to their purity, consisting predominately of the aggregating target protein, they have traditionally been used for refolding studies, in which they served as an easy to separate source of pure target protein (Singh et al. 2015). This long-held misconception has been challenged in recent years as more and more studies have revealed the dynamic, heterogeneous nature of bacterial IBs, which alongside of misfolded protein also contain protein species with amyloid structure as well as native-like and correctly folded protein (Garcia-Fruitos et al. 2005; Park et al. 2012; Jäger et al. 2019a; Jäger et al. 2018; Jäger et al. 2019b; Kloss et al. 2018a, b; Lamm et al. 2020; Zhou et al. 2012; Wang et al. 2015; Jiang et al. 2019; Wu et al. 2011; Lin et al. 2013; Diener et al. 2016; Choi et al. 2011; Nahalka and Nidetzky 2007; Nahalka et al. 2008; Nahalka 2008; Nahalka and Patoprsty 2009; Koszagova et al. 2018; Huang et al. 2013; Arie et al. 2006). Thus, more and more evidence suggests that those properties are to a certain degree an inherent feature of all IBs and that all cytoplasmic proteins exist in a conformational equilibrium between soluble-folded, partially misfolded, and insoluble aggregates. This equilibrium in turn can be shifted depending on certain cellular conditions that favor either soluble production, misfolding, degradation, aggregation as IBs, or disintegration of the latter (Fig. 1a, b). Hereby, it seems reasonable to assume that conditions under which the cellular refolding and degradation machinery is outbalanced (e.g., upon conditions of strong overexpression) favor the formation of IBs. This hypothesis finds further support in recent studies, which have shown that for the same genetic construct, depending on the employed cultivation and induction conditions, either active CatIBs or classical, inactive IBs are formed (Lamm et al. 2020). Here, we refer to IBs that retain a certain degree of catalytic activity (in case of enzymes) or fluorescence (in case of fluorescent reporters) as catalytically active IBs (CatIBs). While anecdotal evidence suggests that proteins and enzymes can form CatIBs naturally (Dong et al. 2014; Garcia-Fruitos et al. 2005; Li et al. 2013; Worrall and Goss 1989; Park et al. 2012; Tokatlidis et al. 1991; Krauss et al. 2017; Nahálka et al. 2006), the majority of studies that reported successful formation of CatIBs relied on molecular biological fusion of a variety of different aggregation-inducing peptides, protein domains, or proteins (Garcia-Fruitos et al. 2005; Park et al. 2012; Jäger et al. 2018; Jäger et al. 2019a, b; Kloss et al. 2018a, b; Lamm et al. 2020; Zhou et al. 2012; Wang et al. 2015; Jiang et al. 2019; Wu et al. 2011; Lin et al. 2013; Diener et al. 2016; Choi et al. 2011; Nahalka and Nidetzky 2007; Nahalka et al. 2008; Nahalka 2008; Nahalka and Patoprsty 2009; Koszagova et al. 2018; Huang et al. 2013; Arie et al. 2006) (Fig. 1c). The resulting CatIBs can thus be considered as cellularly produced, insoluble bionanomaterials, or protein immobilizates (Fig. 1d) with potential application in biocatalysis, synthetic chemistry, and biomedicine (Yang et al. 2018; Jäger et al. 2018; Jäger et al. 2019b; Kloss et al. 2018a, b; Diener et al. 2016; Nahalka 2008; Nahalka and Nidetzky 2007; Nahalka and Patoprsty 2009; Nahalka et al. 2008; Ratera et al. 2014; Rueda et al. 2014; García-Fruitós et al. 2009; Vazquez et al. 2012). Since CatIBs are produced heterologously in bacteria, it is not surprising that different parameters, like fusion protein design, expression conditions, and downstream processing, strongly influence not only the general success of immobilization as CatIBs but also their properties. The latter observation also has direct consequences for biocatalytic application of CatIBs as shown recently in several studies (Jäger et al. 2019a; Kloss et al. 2018a).

With the present mini-review, we present an overview of the CatIB immobilization strategy, to provide some general guidelines for those that want to generate CatIBs for their own biocatalytic needs, at the same time paving the way towards their wider use in biotechnology. To this end, we review general aspects important for the on-demand production of CatIBs such as fusion protein design concepts, suitable molecular biological construction methods, as well upstream and downstream bioprocess parameters and selected recent applications in biotechnology.

## Induction of CatIB formation—suitable tags, target proteins, and optimization strategies

The successful production of CatIBs requires the selection of an aggregation-inducing tag, which has to be fused via suitable linker polypeptides either N- or C-terminally to the target protein/enzyme. This process still requires the testing of various aggregation-inducing tags, fusions sites, and linker polypeptides because a generally applicable strategy does presently not exist. However, from recent studies, some rules can be inferred that might serve as guidelines for fusion protein design. In the following, we will provide an overview of the available aggregation-inducing tags, tested target proteins, and optimization strategies.

### Aggregation-tag selection

**Fig. 2:**
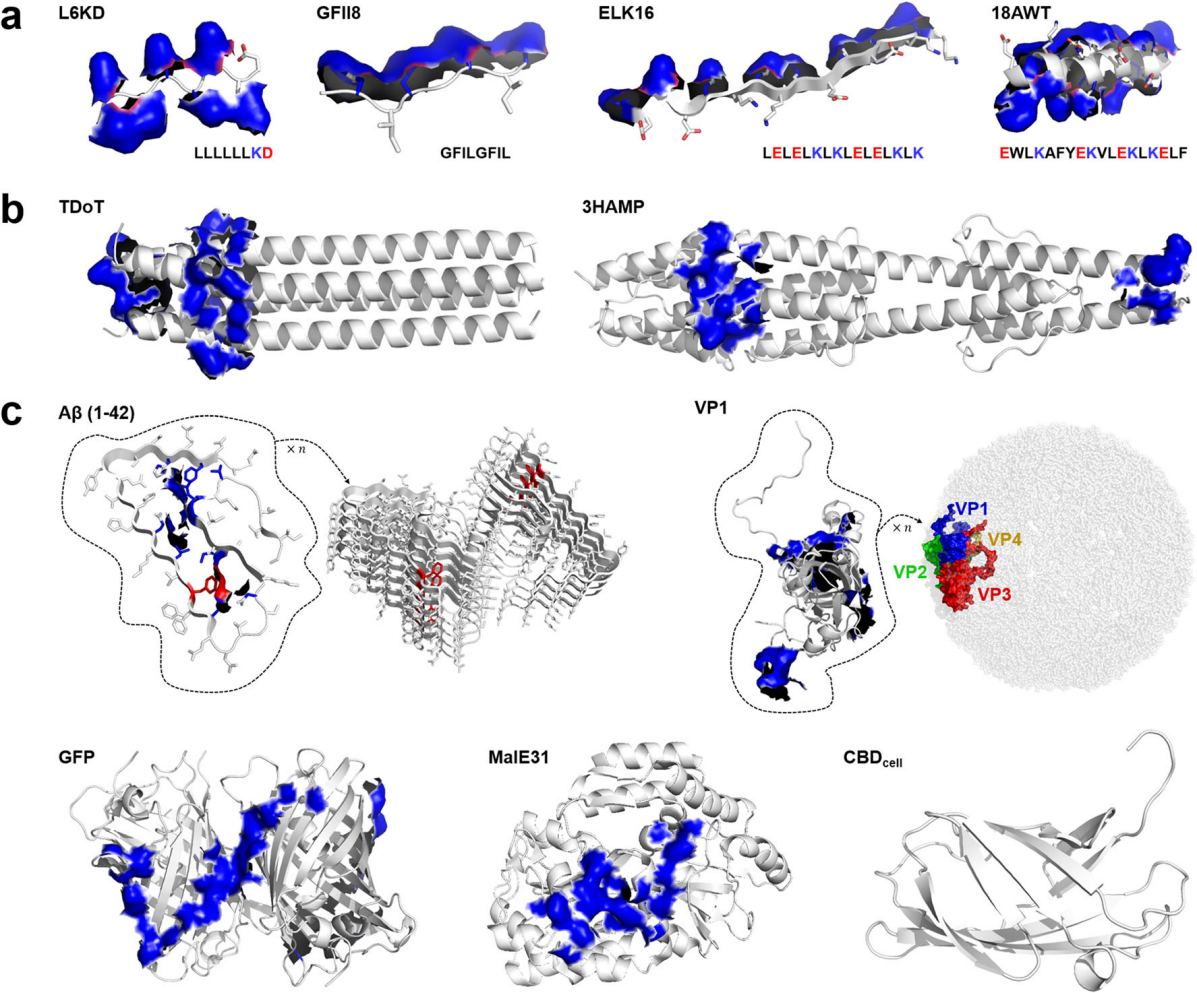
Hydrophobic patch analysis of CatIB formation–inducing tags. All structures are shown in cartoon representation in gray with the Rosetta-identified hydrophobic surface patches shown as blue surfaces (Kuhlman and Baker 2000; Rohl et al. 2004). **a** Artificial peptides: L6KD, GFIL8, ELK16, and 18AWT. Structures were modelled with Yasara (Krieger and Vriend 2014, 2015) to depict their reported structure. Structures are shown in cartoon representation with residues as sticks. Carbon atoms in gray, nitrogen in blue, and oxygen in red. The amino acid sequence (in single-letter code) of each peptide tag is shown below each model, with non-polar residues in black and polar residues in red (anionic residues) and blue (cationic residues), respectively. **b** CatIB formation–inducing coiled coil domains: tetrameric TDoT and dimeric 3HAMP. **c** Aggregation-prone proteins reported to induce CatIB formation. As representative structure of Aβ42 (F19D), the structure of the wild-type Aβ42 monomer is shown (left side; circled with a dashed line) with all side chains in stick representation. F19, residing within the central hydrophobic cluster constituted by residues 17-21 (de Groot et al. 2006), is highlighted in red. In addition, the recently solved structure of the Aβ42 amyloid fibril (Gremer et al. 2017) is shown to illustrate the crossed β-pleated sheet packing of amyloids. For VP1, the foot-and-mouth disease virus (FMDV) capsid protein, the monomeric VP1 subunit (in cartoon representation; circled with a dashed line), as well as the structure of the 240-mer empty capsid constituted by VP1 (blue), VP2 (green), VP3 (red), and VP4 (yellow) of the FMDV A22 (Porta et al. 2013). PDB-IDs: TDoT: 1FE6; 3HAMP: 3LNR; Aβ1-42: 5OQV; VP1: 4IV1; GFP: 1GFL; MalE31: 1LAX; CBDcell: 1EXG. No structures are available for PoxB and CBDclos

Currently, it remains unclear which structural factors, such as polypeptide-chain composition, quaternary structure, or surface composition of the target as well as the tag, dominate the CatIB formation process. Therefore, it is advisable to always test a variety of tags as CatIB-inducing elements, which can differ in size, ranging from small artificial peptides over protein domains up to quite large aggregation-prone proteins. Table 1 summarizes well known and tested CatIB formation–inducing tags, whose structures are depicted in Fig. 2. However, before reviewing the available tags and their properties, we have to address the question: what makes a good CatIB formation–inducing tag? Here, three aspects, which are not totally independent, must be accounted for the following: (i) the CatIB formation efficiency, defined as the activity, or in case of fluorescent proteins, fluorescence, of the insoluble IBs relative to the activity/fluorescence of the crude cell extract, (ii) the yield of the CatIBs, as well as (iii) their residual activity (Jäger et al. 2019a). While the first factor is an indicator for the suitability of the tag to induce CatIB formation, in particular, the last factors are critical for application of CatIBs in biotechnology.

**Table 1:** Overview of different IB-inducing elements. Cases where residual activity was compared only to cell lysate are marked with *. ^1^CatIB formation efficiency: defined as the activity, or in case of fluorescent proteins, fluorescence, of the insoluble IBs relative to the activity/fluorescence of the crude cell extract. ^2^Residual activity compared to purified enzyme. ^3^MenD: 2-succinyl-5-enol-pyruvyl-6-hydroxy-3-cyclohexene-1-carboxylate synthase. ^4^Residual-specific fluorescence compared to cell lysate. ^5^GalU: UDP-glucose pyrophosphorylase. ^6^Volumetric activity. n.i. no information provided. n.a. not applicable

Name (origin)	**Length (no. of amino acids)**	**Tag property**	**Linker/structure**	**Target enzyme/protein**	**Target origin**	**CatIB formation efficiency (%)**^**1**^	**Residual activity (%)**^**2**^	**Ref.**
I. Artificial peptides								
L6KD	8	Amphophilic	PTPPTT PTPPTTPTPTP Unstructured	Lipase A	*B. subtilis*	80	30*	Zhou et al. (2012)
				Amadoriase II	*A. fumigatus*	61	93*	Zhou et al. (2012)
				β-xylosidase	*B. pumilus*	84	26*	Zhou et al. (2012)
				GFP	*A. victoria*	n.i.	n.a.	Zhou et al. (2012)
GFIL8	8	β-sheet	PTPPTT PTPPTTPTPTP Unstructured	Lipase A	*B. subtilis*	89	43*	Wang et al. (2015)
				Amadoriase II	*A. fumigatus*	93	54*	Wang et al. (2015)
				Ulp1 protease	*S. cerevisia*e	n.i.	40*	Jiang et al. (2019)
ELK16	16	β-sheet	PTPPTT PTPPTTPTPTP Unstructured	Amadoriase II	*A. fumigatus*	120	88*	Wu et al. (2011)
				β-xylosidase	*B. pumilus*	94	77*	Wu et al. (2011)
				GFP	*A. victoria*	n.i.	n.a.	Wu et al. (2011)
18A (and variants)	18	α-helical	PTPPTT PTPPTTPTPTP Unstructured	Lipase A	*B. subtilis*	90	150*	Lin et al. (2013)
				Amadoriase II	*A. fumigatus*	n.i.	n.i.	Lin et al. (2013)
				β-xylosidase	*B. pumilus*	n.i.	n.i.	Lin et al. (2013)
				GFP	*A. victoria*	n.i.	n.a.	Lin et al. (2013)
II. Coiled coil domains								
TDoT (*S. marinus*)	53	Tetrameric coiled coil	(GGGS)_3_ Unstructured	Hydroxynitrile lyase	*A. thaliana*	76	11	Diener et al. (2016)
				MenD^3^	*E. coli*	90	n.i.	Diener et al. (2016)
				Lipase A	*B. subtilis*	114	n.i.	Diener et al. (2016)
				Alcohol dehydrogenase	*Ralstonia* sp*.*	88	2	Jäger et al. (2019a); Jäger et al. (2018)
				Alcohol dehydrogenase	*L. brevis*	5	6	Jäger et al. (2019a)
				Benzaldehyde lyase	*P. fluorescens*	88	1	Jäger et al. (2019a); Kloss et al. (2018a)
				Benzoylformate decarboxylase	*P. putida*	1	4	Jäger et al. (2019a)
				Lysine decarboxylase	*E. coli*	n.i.	n.i.	Jäger et al. (2019a); Kloss et al. (2018b)
				YFP	*A. victoria*	65	n.a.	Jäger et al. (2019a); Jäger et al. (2018)
				mCherry	*D. striata*	42	n.a.	Jäger et al. (2019a); Jäger et al. (2018)
3HAMP (*P. aeruginosa*)	172	Dimeric coiled coil	(GGGS)_3_ Unstructured	Alcohol dehydrogenase	*Ralstonia* sp*.*	75	12	Jäger et al. (2019a)
				Alcohol dehydrogenase	*L. brevis*	67	1	Jäger et al. (2019a)
				Benzaldehyde lyase	*P. fluorescens*	76	18	Jäger et al. (2018)
				Benzoylformate decarboxylase	*P. putida*	61	10	Jäger et al. (2019a)
				Lysine decarboxylase	*E. coli*	n.i.	n.i.	Kloss et al. (2018b)
				YFP	*A. victoria*	6	n.a.	Jäger et al. (2019a)
				mCherry	*D. striata*	5	n.a.	Jäger et al. (2019a)
Target (origin)	**Length (no. of amino acids)**	**Tag property**	**Linker/structure**	**Target enzyme/protein**	**Target origin**	**CatIB formation efficiency (%)**^**1**^	**Residual activity (%)**^**2**^	**Ref.**
III. Aggregation-prone proteins (protein domains)								
Aβ42(F19D) (*Homo sapiens*)	42		n.i.	BFP	*A. victoria*	61–65	31^4^	Garcia-Fruitos et al. (2005)
CBDcell (*C. fimi*)	108	Cellulose-binding protein	n.i.	β-glucuronidase	*E. coli*	92	19	Choi et al. (2011)
				β-glycosidase	*T. caldophilus*	93	n.i.	Choi et al. (2011)
				DsRed	*D. striata*	n.i.	n.a.	Choi et al. (2011)
CBDclos (*C. cellulovorans*)	156	Cellulose-binding protein	43 amino acids with thrombin cleavage site, S-Tag™, and cloning site	d-amino acid oxidase	*T. variabilis*	> 90	42*	Nahalka and Nidetzky (2007)
				d-sialic acid aldolase	*E. coli K12*	100	100*	Nahalka et al. (2008)
				Maltodextrin phosphorylase	*P. furiosus*	83	n.i.	Nahalka et al. (2008)
				Cytidylate kinase	*E. coli*	n.i.	n.i.	Nahalka and Patoprsty (2009)
				Polyphosphate kinase PPK3	*S. pomeroyi*	n.i.	n.i.	Nahalka and Patoprsty (2009)
				GFP	*A. victoria*	n.i.	n.i.	Koszagova et al. (2018)
				GalU^5^	*E. coli*	n.i.	n.i.	Koszagova et al. (2018)
VP1 capsid protein (foot-and-mouth disease virus)	209	Virus capsid protein	n.i.	β-galactosidase	*E. coli*	36-46	166*	Garcia-Fruitos et al. (2005)
				GFP	*A. victoria*	n.i.	n.a.	Garcia-Fruitos et al. (2005)
GFP (*A. victoria*)	238	Fluorescent protein	(GGGS)5 Flexible (AAAKE)5 Rigid	Acid phosphatase	*E. aerogenes*	n.i. n.i.	48 58	Huang et al. (2013)
MalE31 (*E. coli*)	396	Maltose binding protein	RIPGG Unstructured	Alkaline phosphatase	*E. coli*	> 95	n.i.	Arie et al. (2006)
				β-lactamase	*E. coli*	> 95	n.i.	Arie et al. (2006)
PoxB (*P. polymyxa* E681)	574	Pyruvate oxidase	n.i.	GFP	*A. victoria*	n.i.	n.a.	Park et al. (2012)
				α-amylase	*B. subtilis*	77	200^6^	Park et al. (2012)

The class of small artificial CatIB-inducing peptide tags shows quite different structural properties: The group of Lin described small β-sheet structures (ELK16 and GFIL8) (Wang et al. 2015; Jiang et al. 2019; Wu et al. 2011) and surfactant-like tags (L6KD) (Zhou et al. 2012), as well as bigger α-helical peptides (18A and variants thereof) (Lin et al. 2013). With these tags, CatIB formation efficiencies between 61 and 120% were achieved and the produced CatIBs showed remarkably high residual activities. However, care should be taken when comparing those values to other studies, as their residual activity was mostly determined relative to the corresponding cell lysate from which they were obtained by centrifugation and not relative to the respective purified target enzyme. An interesting feature of these tags is that they can be used for mild extraction of the at least partially correct folded target from CatIBs without the need for refolding steps (Yang et al. 2018).

Another well-studied group of aggregation-inducing tags used for CatIB production are coiled coil domains. So far, a dimeric (3HAMP: derived from the oxygen sensor protein Aer2 of *Pseudomonas aeruginosa* (Airola et al. 2010)) and a tetrameric coiled coil (TDoT: tetramerization domain of the cell surface protein tetrabrachion of *Staphylothermus marinus* (Stetefeld et al. 2000)) were tested with a broad range of different target enzymes and proteins (Kloss et al. 2018a; Jäger et al. 2018; Diener et al. 2016; Jäger et al. 2019a, b; Kloss et al. 2018b; Lamm et al. 2020). Here, the CatIB formation efficiency was found to differ greatly depending on the target enzyme. In general, the tetrameric TDoT displayed a higher CatIB formation efficiency and yielded CatIBs of a higher purity. However, CatIBs that were produced using the dimeric 3HAMP coiled coil domain as CatIB-inducing tag retained higher residual activity compared to their TDoT counterparts (Jäger et al. 2019a). In addition, 3HAMP CatIBs showed a higher lipid content and a more diffuse structure (as revealed by fluorescence microscopy and scanning electron microscopy), thus indicating a less densely packed structure compared with the corresponding TDoT CatIBs. This in turn could account for their higher residual activity (Jäger et al. 2019a). Notably, the residual activities for coiled coil–induced CatIBs are generally low. However, their residual activity was determined relative to the corresponding purified, soluble enzymes (see above). Furthermore, their recyclability was shown for several targets, in both aqueous and organic-solvent-based reaction systems (Diener et al. 2016; Kloss et al. 2018b). Recently, using those domains, the co-immobilization of two target proteins/enzymes could be demonstrated (Jäger et al. 2018; Jäger et al. 2019b).

In addition to small tags and protein domains, a number of larger proteins and protein domains were tested as CatIB formation–inducing elements. Several of these were selected due to their well-known aggregation tendency, e.g., cellulose-binding domains (CBDs, (Nahalka 2008; Koszagova et al. 2018; Choi et al. 2011; Nahalka and Nidetzky 2007; Nahalka and Patoprsty 2009; Nahalka et al. 2008)). Two different CBDs have been tested for CatIB induction: the rather small 108 amino acid long CBDcell from *Cellulomonas fimi* (Choi et al. 2011), as well as the 156 amino acid long CBDclos from *Clostridium cellulovorans* (Nahalka 2008; Koszagova et al. 2018; Nahalka and Nidetzky 2007; Nahalka and Patoprsty 2009; Nahalka et al. 2008). Most of the CBD-derived CatIBs were only used for proof-of-concept studies and IB formation efficiency, and residual activities were not determined. However, CatIBs of sialic acid aldolase fused to a CBD from *Clostridium cellulovorans* (CBDclos) showed about the same activity as the corresponding soluble protein and could be recycled 19 times without loss of activity (Nahalka et al. 2008). For higher stability and easier recycling, CBD-CatIBs were cross-linked with glutaraldehyde (Nahalka et al. 2008) or magnetized by iron oxide (Koszagova et al. 2018). Furthermore, Aβ42(F19D), a variant of the human Aβ-amyloid peptide and the VP1 capsid protein of the foot-and-mouth disease virus were selected due to their tendency to aggregate (Garcia-Fruitos et al. 2005). Both tags yielded only moderate CatIB formation efficiencies, but in case of VP1, the activity of the resulting β-galactosidase CatIBs could be increased 1.6 times compared with the cell lysate (Garcia-Fruitos et al. 2005). Interestingly, the fluorescent reporter protein GFP from *Aequorea victoria*, which is commonly used as a fusion target and known for its high solubility, can also be used as an aggregation-inducing tag. Here, fusion of GFP to an alkaline phosphatase from *Enterobacter aerogenes* resulted in CatIBs with a residual phosphatase activity of 48 to 58% (Huang et al. 2013). In addition, even larger aggregation-prone proteins have been used for CatIB formation. Those include a variant of the maltose binding protein (MalE31; 396 amino acids) of *E. coli* (Arie et al. 2006) and a pyruvate oxidase (PoxB; 574 amino acids) of *Paenibacillus polymyxa* (Park et al. 2012) that are both significantly bigger than the targets they were fused to. In contrast to most described CatIBs, MalE31-CatIBs could be found in the periplasm, which is the native location of MalE31 (Arie et al. 2006). CatIB induction is hereby likely related to the folding deficiency of the MalE31 variant. PoxB-CatIBs of an amylase showed a twofold higher volumetric activity than the soluble enzyme (Park et al. 2012).

As revealed by this overview, the presently known CatIB formation–inducing elements come in all sizes and show variable secondary, tertiary, and quaternary structures (Table 1). Therefore, it is still not possible to rationally predict the success of the CatIB formation strategy for any combination of tag, linker, and target protein/enzyme. However, first attempts to link CatIB formation and computational aggregation-propensity predictions have been made (Krauss et al. 2017). While no quantitative correlations could be found between the predicted aggregation tendency of the tag, CatIB formation efficiency, and/or CatIB residual activity, all tags were predicted computationally to show the tendency to aggregate with at least one of the employed tools (Krauss et al. 2017). In addition, from the above presented prediction of hydrophobic surface patches (Fig. 2), it becomes apparent that, with the exception of CBDcell, which appears to lack larger hydrophobic surface patches, all CatIB formation–inducing tags possess solvent exposed hydrophobic surfaces, likely contributing to aggregation and hence CatIB formation (see below).

### Target-protein properties

In most proof-of-concept CatIB studies, only model enzymes or even fluorescent proteins were used as targets. Here, a commonly used enzyme was the lipase A from *B. subtilis*, a small (19 kDa), monomeric enzyme that does not require co-factors (van Pouderoyen et al. 2001), as well as the 26 kDa Ulp1 protease from *S. cerevisiae* (Jiang et al. 2019) or the 33 kDa sialic acid aldolase from *E. coli* (Nahalka et al. 2008). Inducing CatIB formation for those rather simple enzymes appears straightforward, as exemplified by relatively high CatIB formation efficiencies (Table 1). However, even larger, more complex oligomeric enzymes, such as the 98 kDa maltodextrin phosphorylase from *P. furiosus* (Nahalka 2008), the homotetrameric β-galactosidase with a total size of 540 kDa (Garcia-Fruitos et al. 2005), and the homodecameric lysine decarboxylase with a total size of 806 kDa (Jäger et al. 2019a; Kloss et al. 2018b), could successfully be produced as CatIBs. Therefore, it seems that size and oligomerization state do not have a predictable impact on the success of CatIB formation. However, it should be noted that all examples reported so far for successful CatIB formation dealt with homo-oligomeric enzymes, as hetero-oligomeric complexes of several catalytic subunits are likely difficult to properly assemble within IBs. In contrast to overall size and quaternary structure, the presence of non-covalently bound co-factors, which need to be recycled during the catalytic cycle, might play a more important role for the activity of CatIBs, since they must not only be correctly bound within the enzyme during CatIB formation but also need to be able to dissociate from and diffuse to the enzyme. However, the present data does not allow unequivocal conclusions in this regard. To this end, Jäger et al. (2019a) empirically compared the production and residual activity of different CatIBs whose production was induced by two different aggregation tags. Here, the highest residual activity was achieved with CatIBs of the only tested enzyme that did not require a co-factor (Table 1; *A. thaliana* hydroxynitrile lyase fused to TDoT), while the same tag yielded only CatIBs with lower residual activity for targets that were co-factor dependent (Table 1; alcohol dehydrogenases of *L. brevis* and *Ralstonia* sp., *P. fluorescens* benzaldehyde lyase, *P. putida* benzoylformate decarboxylase) (Diener et al. 2016; Jäger et al. 2019a; Jäger et al. 2018; Kloss et al. 2018a, b). However, the use of another CatIB formation–inducing tag yielded CatIBs of the same co-factor-dependent enzymes with much higher residual activities (see Table 1; compare TDoT and 3HAMP CatIBs (Jäger et al. 2019a)).

**Fig. 3:**
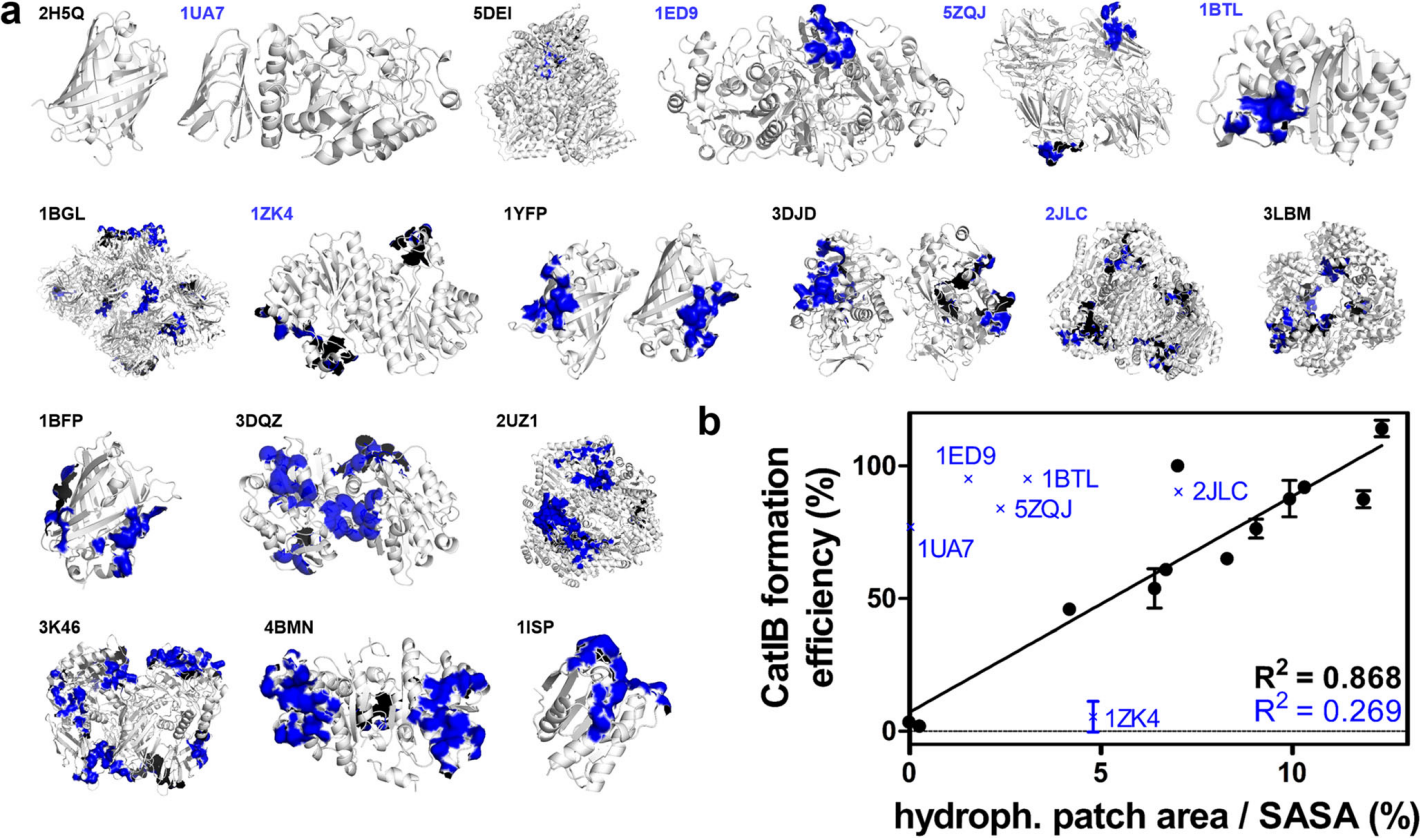
Hydrophobic patch analysis of selected target proteins which were produced as CatIBs. **a** All target proteins from Table 1 for which a structure is known were analyzed for the presence of hydrophobic surface patches. All structures are shown in cartoon representation in gray with the Rosetta-identified hydrophobic surface patches shown as blue surfaces (Kuhlman and Baker 2000; Rohl et al. 2004) calculated as described in Jäger et al. (2019a). Proteins are identified by PDB-IDs (see below). **b** Correlation between CatIB formation efficiency and fraction of hydrophobic surface patches. Hydrophobic surface patches for the corresponding target protein structures were quantified by employing the hpatch tool of the Rosetta modelling suite (Kuhlman and Baker 2000; Rohl et al. 2004; Jacak et al. 2012). Surface areas were quantified using Pymol 1.7.0.0 (Schrödinger, LCC, New York, NY, USA). CatIB formation efficiency as the relative activity of the insoluble CatIB fraction (Table 1). Coefficient of determination values (*R*^2^) is given excluding (black) and including outliers (blue). Outliers are identified by PDB ID and are depicted with blue crosses. PDB-IDs are as follows: 2H5Q: mCherry, 1UA7: *B. subtilis* α-amylase, 5DEI: *P. putida* benzoylformate decarboxylase, 1ED9: *E. coli* alkaline phosphatase, 5ZQJ: *B. pumilus* β-xylosidase, 1BTL: *E. coli* β-lactamase, 1BGL: *E. coli* β-galactosidase, 1ZK4: *L. brevis* alcohol dehydrogenase, 1YFP: yellow fluorescent protein, 3DJD: *A. fumigatus* amadoriase II, 2JLC: *E. coli* MenD, 3LBM: *E. coli*d-sialic acid aldolase, 1BFP: blue fluorescent protein, 3DQZ: *A. thaliana* hydroxynitrile lyase, 2UZI: *P. fluorescens* benzaldehyde lyase, 3K46: *E. coli* β-glucuronidase, 4BMN: *Ralstonia* sp. alcohol dehydrogenase, 1ISP: *B. subtilis* lipase A

Thus, size, oligomerization state, and co-factor dependency do not appear to be decisive or limiting factors for CatIB formation. Given the structural diversity of the so far employed target proteins, the question arises, if there are any mutual structural features that are important for CatIB formation. To the best of our knowledge, the only information, although limited in scope due to the small size of the dataset, again comes from Jäger et al. who showed that target enzymes possessing larger hydrophobic surface patches (Fig. 3) generally displayed higher CatIB formation efficiencies (Jäger et al. 2019a). This suggests that CatIB formation not only is driven by the aggregation-inducing tag but, at least to a certain extent, also depends on the interactions of the target proteins (and/or the tag) caused by the physical proximity of the target molecules themselves. This is illustrated by the observation that the CatIB formation efficiency observed for TDoT-CatIBs of mCherry was much reduced as compared with the corresponding YFP TDoT-CatIBs (Jäger et al. 2019a), which might be related to the fact that monomeric mCherry virtually lacks any hydrophobic surface patches compared with dimeric YFP (Fig. 3a; compare mCherry: 2H5Q; eYFP: 1YFP). Please note that a correlation between hydrophobic patch area and CatIB formation efficiency (Fig. 3b) only holds for 12 out of 18 of the here analyzed targets. For five targets, high CatIB formation efficiencies but only moderate hydrophobic patch areas are observed, while only one target (Fig. 3b; 1ZK4) shows moderately large hydrophobic patches but only low CatIB formation efficiency. While this analysis apparently does not allow for precise prediction of the CatIB formation efficiency based on structure, hydrophobic surface patches nevertheless seem to play an important role for the process.

### Optimization strategies—fusion sites and linkers

From a structural perspective, several factors need to be considered when genetic fusions are designed to induce CatIB formation. First of all, a fused tag should not interfere with correct folding of the enzyme to its catalytically active form. Hence, apart from the overall monomeric structure, also the enzymes’ native quaternary structure needs to be considered when designing the fusion construct. This was for example demonstrated for the lysine decarboxylase from *E. coli*, where the N-terminus is buried within the decameric structure of the enzyme, while the C-terminus is located at the protein surface. In accordance, the activity of the CatIBs derived from C-terminal fusion of TDoT was about six orders of magnitude higher than for the corresponding N-terminal fusion (Jäger et al. 2019a; Kloss et al. 2018b). Thus, in conclusion, the fusion site (N- vs C-terminal) should be carefully evaluated and if no structures are available, both sites need to be tested.

Another factor that can influence the success of CatIB formation is the presence and nature of polypeptide linkers that are employed to link the CatIB formation–inducing tag and the target enzyme. These linkers can differ greatly in size and function, e.g., flexible vs rigid linker motifs (Table 1). For GFP-induced CatIBs, the effect of the linker with regard to the aggregation propensity has been studied. Here, the exchange of the flexible (GGGS)_5_-linker to the rigid (AAAKE)_5_-linker improved the residual activity of the target enzyme by about 10% (Huang et al. 2013). Interestingly, this is reminiscent of a different study, where the deletion of the flexible (GGGS)_3_-linker enhanced the CatIB formation efficiency of TDoT-mCherry CatIBs by about 30% (Jäger et al. 2019a). Other studies utilize protease cleavage sites as linkers in order to analyze CatIB fusion and target enzyme independently (Nahalka 2008; Nahalka and Nidetzky 2007; Nahalka and Patoprsty 2009; Nahalka et al. 2008; Koszagova et al. 2018). CatIBs induced by artificial peptides always contained a flexible 17 amino acid proline-threonine linker of about the same length as the aggregation tag. However, its function is not discussed in the studies (Jiang et al. 2019; Lin et al. 2013; Wang et al. 2015; Wu et al. 2011; Zhou et al. 2012).

In conclusion, the design of fusion proteins for CatIB formation is presently still a trial-and-error process and requires testing of multiple constructs, e.g., different CatIB formation–inducing tags, different fusions sites, and different linker polypeptides. Therefore, one limiting factor for the CatIB approach is the cloning strategy used for fusion construct design, which will therefore be reviewed in the following.

### Towards automated fusion-protein generation—high-throughput cloning, expression, and hit identification

The construction of fusion proteins for CatIB production is usually performed by traditional cloning with classical restriction enzymes (Arie et al. 2006; Choi et al. 2011; Diener et al. 2016; Garcia-Fruitos et al. 2005; Jiang et al. 2019; Lin et al. 2013; Wang et al. 2015; Wu et al. 2011; Zhou et al. 2012); however, this is not convenient to generate an extensive CatIB library of larger numbers of variants due to numerous laborious steps. In a few cases, already more generic and concomitantly less time-consuming cloning methods like LICing and Gibson Assembly were successfully used for gene fusion generation (Heater et al. 2018; Nahalka 2008; Nahalka and Nidetzky 2007; Nahalka and Patoprsty 2009). Using modern cloning methods is a major step towards the generation and screening of a CatIB library to find the best CatIB variant in less time.

For example, Nahálka and colleagues applied ligase independent cloning (LICing) for the production of CatIBs (Nahalka 2008; Nahalka and Nidetzky 2007; Nahalka and Patoprsty 2009). The advantage of this method is that no restriction enzymes and T4 DNA ligase are needed. The linearized vector and insert are treated with T4 polymerase, due to its 3′ ➔ 5′ exonuclease activity, and only one kind of nucleotide triphosphate is added. Removing nucleotides from the 3′-end lead to single-stranded DNA tails, which are formed until the first complementary base of the added nucleotide triphosphate is reached. Due to the designed complementarity of the treated vector and insert, the cohesive ends of the DNA fragments anneal to form a plasmid that can be used for transformation of bacteria (Aslanidis and Dejong 1990). An advancement of LICing is PLICing, phosphorothioate-based ligase-independent gene cloning, which was developed in 2010 by Blanusa and co-workers (Blanusa et al. 2010). In comparison to traditional LICing, the advantage is that no enzyme, no gel extraction and no purification are needed. First, the vector and the target gene are amplified via PCR with specific primers that have complementary phosphorothioate nucleotides at the 5‘-end. After amplification, the PCR products are treated with an iodine/ethanol solution, which cleaves phosphorothioate bonds, producing single-stranded DNA tails. Finally, the vector and the target gene are hybridized to generate a circular plasmid that can be used to transform competent cells. In comparison to traditional LICing, with PLICing, also large DNA fragments (>6 kb) can be formed, which could be beneficial for larger combinations of enzyme and aggregation tag. Moreover, the cleaved fragments do not have to be purified, which is a time saving benefit (Blanusa et al. 2010). In-Fusion™ assembly is a further cloning method without the use of a ligase. A seamless cloning can be achieved by using DNA fragments with the same 15 bp overlaps that can be assembled after the DNA polymerase of poxvirus with its 3′-5′ proofreading activity has removed nucleotides from the 3′ end. The complementary nucleotides can join and form a combined DNA molecule. *E. coli* will repair the remaining small gaps in the molecule after transformation (Zhu et al. 2007).

With Gibson Assembly, Heater and co-workers used another modern cloning technique (Heater et al. 2018). Their fusion constructs consisted of a GS-Linker, a Cry3Aa Tag, and the respective gene of interest. The resulting fusion protein formed solid, crystal-like particles in *Bacillus thuringiensis*, which possess a certain morphological similarity to CatIBs, and have hence been included here. Gibson Assembly as an isothermal, single-reaction method, enabling multiple DNA fragments to be joined during a PCR if they have matching overhangs. To achieve this, three different enzymes are needed: a 5′ exonuclease, a Phusion DNA polymerase, and a *Taq* DNA ligase. First, a 5′ exonuclease generates single-stranded DNA overhangs by removing nucleotides from the 5′ ends of the double-stranded DNA fragments. Complementary single-stranded DNA overhangs can anneal, and the Phusion DNA polymerase is able to fill the gaps. Finally, the *Taq* DNA ligase can seal the nicks, and joined, double-stranded DNA molecules are generated (Gibson et al. 2009).

An additional alternative cloning technique well suited for CatIB library generation is Golden Gate cloning. It is characterized by the use of a type II restriction enzyme, which is able to cleave DNA outside of its recognition site. After restriction digestion, the recognition site is cut out of the desired fragment and a four-nucleotide overhang is generated, which can be ligated with the matching DNA overhang from the next fragment. The whole reaction can take place in a so-called one-pot setup, because ligation and restriction digest are performed at the same time (Engler et al. 2008). Thus, Golden Gate cloning could be the most efficient method for CatIB library generation, since the three different DNA elements can be assembled in an effortless manner with pipetting all elements as the only time-consuming part. However, this can be easily performed by lab automation technology, which could be seamlessly hyphenated with the next steps like the transformation of an expression host with the Golden Gate products, CatIB production, and CatIB purification and analysis. The general automation of such molecular biology workflows have been successfully demonstrated for *E. coli* (Ben Yehezkel et al. 2011; Billeci et al. 2016; Olieric et al. 2010), which is the current major producer of IBs (Carrio et al. 1998; Ventura and Villaverde 2006) as well as CatIBs in literature. The automation of all these processes would be desirable to enable fast provision of suitable CatIBs for new catalytic enzymes.

## Bioprocess development for CatIB production—upstream and downstream considerations

For the development of a bioprocess to efficiently produce and isolate CatIBs, various special characteristics of CatIBs have to be considered to obtain high amounts of highly active CatIBs. Conventionally, either properly folded, active, and soluble proteins or misfolded, inactive aggregated, and insoluble IBs are produced. Since CatIBs not only consist of a scaffold of misfolded aggregated protein, but also contain active, correctly folded or native-like protein species, the characteristics of properly folded as well as aggregated insoluble proteins have to be considered for efficient CatIB production and isolation. In this section, the literature on conventional IBs and CatIBs is reviewed with respect to upstream and downstream process development.

### Important process parameters for CatIB production

For upstream process development, conditions have to be selected that yield active and mostly aggregated proteins, as soluble proteins will be discarded with the soluble cell fraction during CatIB isolation. Therefore, heterologous host selection and cultivation conditions are critical to yield a high IB formation efficiency with high amounts of properly folded and therefore active proteins.

As for conventional IBs, temperature is the most studied cultivation parameter for CatIBs production. Generally, lower cultivation temperatures lead to CatIBs with higher activity (de Groot and Ventura 2006; Doglia et al. 2008; Jevsevar et al. 2005; Peternel et al. 2008; Lamm et al. 2020; Vera et al. 2007; Wang et al. 2017; Arie et al. 2006), whereby lower temperature likely results in the production of a larger fraction of properly folded protein that is incorporated within the CatIB matrix. While CatIBs with higher activities can be produced at lower cultivation temperature, different studies show that lower amounts of CatIBs or less stable CatIBs are produced under those conditions (de Groot and Ventura 2006; Peternel et al. 2008; Doglia et al. 2008). Although, a lower stability can be beneficial to obtain active, soluble proteins from conventional IBs, CatIBs with higher stability would be preferable for application as reused or immobilized biocatalysts (Krauss et al. 2017). Otherwise, the desired product can be contaminated by solubilized protein derived from disintegrating CatIBs during biocatalysis. Thus, for CatIB production, a cultivation temperature has to be chosen or empirically identified that is optimal for yielding high amounts of highly active and stable CatIBs. Here, often a compromise between yield and activity is necessary.

Another cultivation parameter that also strongly influences the production of conventional, inactive IBs is the induction strength. For conventional, inactive IBs, lower induction strength leads to less IBs and more soluble and active proteins (Jhamb and Sahoo 2012; Margreiter et al. 2008). For CatIBs, it was also shown that more active proteins are produced at lower induction strength. However, the amount of active proteins in IBs was decreased so strongly that a higher induction strength leads to an overall higher activity in CatIBs (Lamm et al. 2020). Possibly, misfolded proteins enhance the aggregation of correctly folded proteins, which leads to higher amounts of CatIBs with correctly folded protein. To identify the best induction conditions, *E. coli* Tuner rather than BL21(DE3), which is most frequently used for CatIB production, could be used as expression host to finely adjust the induction strength by inductor dosing (e.g., isopropyl β-d-thiogalactopyranoside; IPTG). For eukaryotic proteins, the host *E. coli* Rosetta might be beneficial as it was superior for the production of a human oxidase as CatIBs compared with the BL21(DE3) host (Wang et al. 2017). For the production of food-grade or pharmaceutically relevant biologics, the need for downstream endotoxin removal can complicate the production process. Thus, the use of expression host strains that lack either endotoxic lipopolysaccharide (LPS) such as *Lactococcus lactis* (Song et al. 2017) or *E. coli* strains that contain genetically modified LPS (Mamat et al. 2015) would be favorable. Both hosts have recently been used for the production of CatIBs (Gifre-Renom et al. 2018; Cano-Garrido et al. 2016) or IBs (Viranaicken et al. 2017).

Regarding the impact of oxygen availability during cultivation, no conclusion can be drawn yet for CatIB production, as its role was hardly studied or no general trend could be observed (Lamm et al. 2020; Worrall and Goss 1989). Similarly, the impact of the growth medium on CatIB production has not been studied thoroughly. While CatIBs are mostly produced in complex media, it was shown that they can also be produced in mineral media (Lamm et al. 2020). However, as the choice of the cultivation medium and supplementations of salts, vitamins, and amino acids have a complex impact on *E. coli*’s metabolism, no general recommendations can yet be given for CatIB production (Hoffmann et al. 2004; Li et al. 2014).

Due to the solid, amorphous nature of CatIB immobilizates, diffusional limitation of educts and products to/from CatIBs during biocatalytic reactions is certainly an issue (Diener et al. 2016). Therefore, CatIB size might be an important parameter for CatIB application. As shown by Kopp et al. (2018) for conventional, inactive IBs, the size of IBs can be adjusted by nutrient feeding. This strategy might also be applicable for CatIB production to optimize the specific activity of CatIBs.

In conclusion, for CatIB production, the cultivation temperature and induction strength had the strongest impact on CatIB productivity. As both parameters strongly influence the protein synthesis rate, both parameters should be investigated simultaneously in small-scale cultivations. Therefore, the BioLector technology in combination with a high-throughput temperature profiling system could be used (Kunze et al. 2014; Samorski et al. 2005). It is important to note that with the same genetic construct CatIBs, mostly soluble proteins or conventional, inactive IBs can be produced by changing a single cultivation parameter (Lamm et al. 2020). Therefore, it might be necessary to screen the expression conditions for potential new CatIB constructs within a certain process window, e.g., by profiling expression temperature, inductor concentration, and induction time, as simulation of protein folding and aggregation with fusion proteins is at present not feasible (Krauss et al. 2017; Lamm et al. 2020; Jäger et al. 2019a; Huber et al. 2009).

### Important process parameters for CatIB purification

For conventional, inactive IBs that are commonly used as starting material for protein renaturation, methods for lab and production scales have been developed (Vallejo and Rinas 2004). While low-speed centrifugation of cells is often followed by a chemical-enzymatic cell lysis step in microliter scale, at larger scales, it is followed by mechanical cell disruption. Those protocols are already applied for CatIB purification at small scales. Even for CatIB production at large scale, protocols for the isolation of conventional, inactive IBs could be applied. However, two major differences may have to be considered for CatIB purification compared with conventional IBs.

First, the stability of CatIBs might be lower as cultivation conditions are applied that promote correct protein folding. As discussed above, this might lead to a decreased CatIB stability that could lead to CatIB disintegration during purification. Those CatIB properties have also been exploited for the purification of soluble protein by solubilization under mild, non-denaturing conditions employing mild detergents at low concentration (Peternel et al. 2008). Therefore, CatIB stability should be monitored during upstream and downstream process development.

Secondly, CatIB preparations might have higher purity requirements due to their application compared with conventional, inactive IBs, which are often contaminated with bacteria by incomplete cell lysis. For small-scale purification, this requirement was already addressed by Rodriguez-Carmona et al. (2010), who developed a protocol which included cell lysis by sonication, multiple enzymatic treatment, and detergent washing steps. However, this protocol might not be economically viable for large-production processes due to high costs for multiple enzymatic purification steps (Vallejo and Rinas 2004).

### Special considerations for the analysis of CatIB activities, purities, and yields

Due to the insoluble nature of the CatIBs, a few additional factors and limitations have to be considered for the optimization of the production process, i.e., compared with the production of soluble enzymes or the use of carrier immobilized enzymes (Mestrom et al. 2020; Francis and Page 2010; Zerbs et al. 2014). For CatIB production, the determination of important quality parameters such as yield, activity, and stability is complicated by the particulate nature of the CatIB material. Overall, three major aspects have to be considered when working with CatIBs.

First of all, the determination of CatIB activities is difficult, as common colorimetric/fluorometric assays for the determination of enzyme activity are usually designed for soluble enzymes thus working in optically transparent (non-turbid) samples. Therefore, methods need to be employed that are suitable for turbid solutions, i.e., reducing the problem of light scattering and reabsorption, i.e., in fluorometry. One solution that helps to address this issue is the use of fluorescence spectrophotometers that enable measurement in a so-called front-face geometry (Eisinger and Flores 1979). Here, the excitation light is focused on the front surface of the cuvette, and fluorescence emission is recorded at an angle of, e.g., 45°, to mitigate the impact of light scattering. This technique is superior for turbid samples such as CatIBs (Jäger et al. 2019a). Alternatively, common colorimetric, absorbance-based assays can be used, when the particulate material is removed, e.g., by centrifugation, before an optical measurement is performed (Jäger et al. 2019b). This, however, complicates the measurement of initial rate velocities, as assay solutions have to be sampled rapidly after the initiation of the reaction. In addition, methods need to be established that rapidly stop the enzymatic reaction before the centrifugation step, which can be achieved by adding denaturing solutions to the assay sample (Jäger et al. 2019b). Alternatively, optical methods can be avoided altogether by, e.g., switching to high-performance liquid chromatography (HPLC)- or gas chromatography (GC)-based methods to monitor product formation or substrate consumption (Diener et al. 2016; Jäger et al. 2019a, b). Those, however, still require rapid termination and sampling as well as the removal of the particulate material. Such methods are therefore hardly adaptable for high-throughput screening purposes or process development.

Secondly, common assays for the determination of protein concentration were also developed for optically transparent (non-turbid) samples. While special adaptions of, e.g., the Bradford assay (Bradford 1976) exist for turbid samples (Gotham et al. 1988), which could be employed for CatIBs, in our hands, those methods proved error prone and less reliable. Therefore, we usually rely on the solubilization of freeze-dried CatIBs in 6M guanidinium chloride solution followed by measuring the protein absorbance at 280 nm. Please note that this method tends to be less precise, when the target protein only represents a smaller fraction of the insoluble CatIB material and fails to account for other proteinaceous impurities and nucleic acid contaminations that have been observed to be present in certain inclusion body preparations (Kloss et al. 2018a; Neerathilingam et al. 2014).

Last but not least, as with other enzyme immobilizates, also CatIBs might be prone to diffusional limitations (Diener et al. 2016; Mestrom et al. 2020), which can further complicate the determination of CatIB activities, i.e., compared with the activity of the same soluble enzyme. Due to those facts, we believe that CatIB activities in many cases have rather been underestimated (not considering other impurities and diffusional limitation).

## Biotechnological potential and application of CatIBs

Last but not least, we will briefly outline the application potential for CatIBs for biocatalysis, synthetic chemistry, and biotechnology. Here, we will not focus on biomedical applications of CatIBs or IBs as this aspect has been reviewed recently (Ratera et al. 2014; Krauss et al. 2017).

The use of enzymes in biocatalysis, biotechnology, and synthetic chemistry, especially in an industrial setting, often requires harsh reaction conditions such as high temperatures, extreme basic or acidic pH values, or the use of organic solvents (Castro and Knubovets 2003; Sheldon and Brady 2018). Therefore, after (heterologous) production, enzymes are often immobilized in or on carrier materials, which in many cases results in a more stable enzyme formulation, while at the same time allowing for easier catalyst handling and recycling (Sheldon and Brady 2018, 2019; Sheldon and van Pelt 2013). At present, the immobilization process, i.e., the selection of appropriate methods and carrier materials has still to be optimized on a case to case basis for each new enzyme. Thus, apart from enzyme production and purification, immobilization represents a major cost and labor factor (Tufvesson et al. 2011) that limits the widespread industrial application of enzymes in synthetic applications. The use of CatIBs could circumvent those problems, as CatIBs essentially represent a fast and economical approach to produce enzyme/protein immobilizates.

To illustrate their utility, various CatIBs have been analyzed with regard to recyclability (Nahalka 2008; Nahalka et al. 2008; Koszagova et al. 2018; Choi et al. 2011; Jiang et al. 2019; Diener et al. 2016; Kloss et al. 2018b). For example, CBDclos-CatIBs with maltodextrin phosphorylase (Nahalka 2008), sialic acid aldolase (Nahalka et al. 2008), or UDP-glucose pyrophosphorylase (Koszagova et al. 2018) could all be recycled for 10 or more times without less than 10% activity loss. However, some of them were further immobilized by alginate (Nahalka et al. 2008) or magnetization (Koszagova et al. 2018), to allow for easier handling and separation. CBDcell-CatIBs with β-glucuronidase or β-glycosidase were both further stabilized by cross linking with glutaraldehyde, displaying no activity loss after three reaction cycles, while without cross-linking the CatIBs lost 65 and 35% of their activity, respectively (Choi et al. 2011). However, in most examples, recycling was tested in aqueous buffer systems. The influence of organic additives was tested with coiled coil–induced CatIBs: TDoT-CatIBs of the thiamine diphosphate (ThDP)-dependent enzyme MenD of *E. coli* showed about 90% activity after 8-time recycling in a buffer containing 5% methyl-*tert* butyl ether (MTBE) (Diener et al. 2016), while TDoT-CatIBs of the *A. thaliana* hydroxynitrile lyase (HNL) did not lose activity after five reaction cycles in a microaqueous system containing almost solely MTBE (Diener et al. 2016).

Another issue that was analyzed repeatedly is the stability and activity of CatIBs. Here, e.g., the stability of the benzaldehyde lyase of *P. fluorescens* (*Pf*BAL) could be considerably enhanced by the immobilization in TDoT-CatIBs (Kloss et al. 2018a). In addition, 3HAMP-CatIBs of *Pf*BAL proved useful in a biphasic system with 70% CPME, in which they showed 3 times higher activity than the corresponding soluble enzyme (Kloss et al. 2018a). GFIL8-CatIBs of the protease Ulp1 showed less leakage after 8 days of repeated recycling and storage compared to immobilizates produced by affinity binding to a cellulosic carrier via a fused cellulose-binding module CBM-tag (Jiang et al. 2019). TDoT-HNL CatIBs were significantly more stable at acidic pH values than their soluble counterpart; the half-life at pH 4.5 was with 290 min more than 100 times longer than for soluble HNL (Diener et al. 2016).

While most CatIBs studies do not go beyond mere proof of concept, e.g., illustrating the general feasibility if the fusion strategy yields CatIBs, a few examples exist where CatIBs have been used for synthetic purposes. One such example for a CatIB-based application is the biosynthesis of 1,5-diaminopentane (also known as cadaverine), a precursor for the production of bio-based polyamides. Here, CatIBs of a constitutive lysine decarboxylase (LDC) of *E. coli* were used to convert l-lysine, which was produced by whole-cell fermentation of a suitable *Corynebacterium glutamicum* strain to cadaverine (Kloss et al. 2018b). The process was tested in batch and repetitive batch mode for up to 69 h of total reaction time and could well compete with other reported approaches that used immobilized LDC in whole cells (Oh et al. 2015; Kind et al. 2014), alginate immobilizates (Bhatia et al. 2015), or cross-linked enzyme aggregates (CLEAs) (Park et al. 2017). Another recent application focused on the co-immobilization of two different enzymes within the same IB-particle in order to realize a CatIB-based synthetic reaction cascade (Jäger et al. 2018; Jäger et al. 2019b). For this purpose, an alcohol dehydrogenase from *Ralstonia* sp. (*R*ADH) and *Pf*BAL was utilized to achieve the synthesis of (1*R*,2*R*)-1-phenylpropane-1,2-diol, an enantiopure 1,2-diol, that represents a building block for different pharmaceuticals and chemicals. In the resulting recycling cascade, encompassing two enzymatic steps and co-substrate coupled recycling of the nicotine amide co-factor of the *R*ADH, CatIBs as well as Co-CatIBs greatly outperformed the soluble enzymes, which were shown to be related to an increase in stability for the (Co)CatIBs (Jäger et al. 2019b). The later example also shows that co-factor recycling is generally possible in CatIBs, although also here diffusional limitation might be a severe problem that limits productivity. Hence, further studies would be needed that address this important issue in more detail.

Last but not least, although not directly related to biocatalysis and synthetic chemistry, the use of CatIBs for mild protein extraction should be mentioned. Even though catalytic activity of the employed IB does not play a direct role here, the same aggregation-inducing tags as used for CatIB formation are used to produce IBs containing a (partially) correctly folded target protein. The use of CatIBs as protein source hereby greatly simplifies the production/purification of the target, by rendering solubilization and refolding steps obsolete (Yang et al. 2018). This technique was tested in several approaches with small artificial peptides as aggregation-inducing tags (ELK16 (Xu et al. 2016; Zhao et al. 2017; Zhao et al. 2016) and L6KD (Zhao et al. 2017)). Here, the authors genetically fused a self-cleaving Mxe GyrA intein between target and IB-inducing tag to enable autocatalytic cleavage of the tag and subsequent release of the target from the IB. With this method, they successfully produced a set of small peptides that are normally unstable and susceptible to proteolytic degradation within bacteria.

## Conclusions and future perspectives

By now, a wealth of examples exists demonstrating the successful induction of CatIB formation with targets covering a broad spectrum of differently complex proteins from simple monomeric fluorescent reporter proteins to complex oligomeric co-factor–dependent enzymes. These data clearly suggest that the CatIB strategy is generically, or at least widely, applicable. Importantly, optimization strategies and a target/tag-centered rationale for the CatIB formation process have been brought forward in recent years, suggesting that the on-demand production of CatIBs for any given target protein might be within reach. From those studies, the following guidelines and future perspectives can be inferred:

- The selection of the fusion terminus for attachment of the CatIB-inducing tag needs careful consideration, e.g., with regard to accessibility based on the quaternary structure of the target protein (Jäger et al. 2019a).
- The choice of linkers (rigid vs flexible) or the lack of a linker is important for the success of the strategy and represents an important optimization strategy (Jäger et al. 2019a).
- The use of short artificial peptide tags to induce CatIB formation appears advantageous as often higher residual activities were observed (Jäger et al. 2019a; Wang et al. 2015; Wu et al. 2011; Zhou et al. 2012). However, empirical comparative studies using more complex target proteins are needed to truly assess their usefulness.
- The presence of aggregation-prone sequence motifs and of hydrophobic surface patches on tag and target might be an important factor influencing CatIB formation for certain targets (Jäger et al. 2019a; Krauss et al. 2017).
- At present, the success of the CatIB strategy for a given target protein cannot be predicted. Therefore, high-throughput experimentation, including high-throughput cloning, e.g., relying on modern restriction enzyme free approaches, as well as automated imaging, would be needed to speed up construct generation and validation.
- More and more successful CatIB application examples and datasets that become available may allow for developing data-driven optimization algorithms or even machine learning algorithms which can lead to hypothesis generation about the structure function relationships required for successful CatIB formation. With this, even the rational design of CatIBs from scratch might become feasible in the years to come.
- For upstream process development, it is crucial to identify whether the catalytic activity is reaction- or diffusion-limited. Depending on the results, the respective bioprocess should be adjusted to produce smaller CatIBs to achieve an increase in specific CatIB activity.
- Identification of culture conditions that yield not only high amounts of correctly folded proteins (e.g., low induction strength, low temperature) but also high amounts of CatIBs (high induction strength) is instrumental for success (Lamm et al. 2020).
- Alternative purification strategies, e.g., relying on magnetization or the use of synthetic biology tools for cell lysis (Pasotti et al. 2011; Koszagova et al. 2018), could speed up CatIB isolation and purification, which would render the associated process more economic.

In conclusion, we believe that CatIBs, as novel, biologically produced enzyme immobilizates possess broad application potential in biocatalysis, synthetic chemistry, and industrial biotechnology. In particular, due to their simple and inexpensive production, CatIB-based enzyme immobilizates and the corresponding technologies contribute to the sustainable management of resources in a bioeconomic setting.

## Data Availability

Not applicable.
